# Insights into the impact on daily life of the COVID-19 pandemic and effective coping strategies from free-text analysis of people's collective experiences

**DOI:** 10.1098/rsfs.2021.0051

**Published:** 2021-10-12

**Authors:** Adam Hampshire, Peter J. Hellyer, William Trender, Samuel R. Chamberlain

**Affiliations:** ^1^ Department of Brain Sciences, Imperial College London, UK; ^2^ Institute of Psychiatry, Psychology and Neuroscience, King's College London, UK; ^3^ Department of Psychiatry, Faculty of Medicine, University of Southampton, UK; ^4^ Southern Health NHS Foundation Trust, UK

**Keywords:** pandemic, COVID-19, mental health, free text, topic modelling, pragmatic interventions

## Abstract

There has been considerable speculation regarding how people cope during the COVID-19 pandemic; however, surveys requiring selection from prespecified answers are limited by researcher views and may overlook the most effective measures. Here, we apply an unbiased approach that learns from people's collective lived experiences through the application of natural-language processing of their free-text reports. At the peak of the first lockdown in the United Kingdom, 51 113 individuals provided free-text responses regarding self-perceived positive and negative impact of the pandemic, as well as the practical measures they had found helpful during this period. Latent Dirichlet Allocation identified, in an unconstrained data-driven manner, the most common impact and advice topics. We report that six negative topics and seven positive topics are optimal for capturing the different ways people reported being affected by the pandemic. Forty-five topics were required to optimally summarize the practical coping strategies that they recommended. General linear modelling showed that the prevalence of these topics covaried substantially with age. We propose that a wealth of coping measures may be distilled from the lived experiences of the general population. These may inform feasible individually tailored digital interventions that have relevance during and beyond the pandemic.

## 1. Introduction

The coronavirus disease 2019 (COVID-19) pandemic has led to unprecedented changes in people's daily lives, with implications for mental health and well-being [1–4], both at the level of a given country's population, and when considering specific vulnerable groups [5–7]. In order to mitigate the untoward impact of the pandemic (including lockdown) and support mental health, it is necessary to identify coping measures that are effective and that people can implement. Indeed, international and national agencies including the World Health Organization, the National Health Service (NHS, UK), Royal College of Psychiatrists (RCPsych, UK), and Centers for Disease Control and Prevention (USA) have provided practical advice for members of the public [8–11]. However, due to the rapidity with which the crisis developed, and limited data from previous pandemics [6], much of this advice is based on expert opinion rather than ‘hard data’. There also is a tendency towards a ‘one size fits all’ approach: that is, assuming that a small set of strategies will be relevant to people from diverse backgrounds. Counter to this view, our analyses of citizen science data, collected from hundreds of thousands of UK residents during 2020 [12–15], show that people have been affected in different ways and that these idiosyncrasies covary substantially with population demographic variables; a corollary of this is that people from different sub-populations are also likely to find different advice useful.

Most relevantly, in a recent study, we observed that the ways in which people's daily lives had been affected markedly differed across age groups, with older adults tending to report greater increases in anxiety and depression levels, and heightened concerns about their health, whereas younger adults were more likely to report disrupted lifestyle and teenagers were more likely to report increased conflict at home. These findings reinforce the message advocated in a recent position statement [5] and elsewhere [16,17] that there is cause for concern about the mental health of both younger and older people during the pandemic, but highlight that the ways age groups have been affected also is quite distinct.

Another example is that of people with pre-existing mental health conditions, who were more likely to show the negative impact of the pandemic on their daily lives [12], but in different ways depending on whether they had depression, anxiety, attention deficit hyperactivity disorder or obsessive–compulsive disorder [12,18–21]. Relatedly, people who were severely ill with COVID-19 had a higher probability of post-traumatic stress disorder [13]. Furthermore, work and home context variables, such as working in healthcare [7,22], being furloughed, becoming unemployed, cohabiting with young children, and having access to green spaces, all had differential relationships with both symptoms of mental health and the ways in which daily lives had been disrupted during the pandemic [12]. Given these findings of the highly idiosyncratic impact of the COVID-19 pandemic on daily life and dimensions of mental health, it is likely that approaches to mental health interventions must be tailored based on a given person's profile.

Conducting surveys using prespecified questions and answers to quantify the pandemic impact and identify practical coping measures is the most commonly used research approach. However, inherent in this methodology is potential for bias towards the views of the surveyor. Such approaches are prone to overlooking the key topics that are most relevant to the general population. A powerful means of addressing these limitations is to consider the general population as a large-scale expert panel and to learn from their collective experiences of the COVID-19 pandemic. To achieve this, people can be asked to express their experiences using free text, collected online and at a large scale. It is not feasible for surveyors to read an entire large corpus of text, and doing so would again risk bias due to their expectations regarding what the common topics should be. Therefore, the optimal solution to this problem is to apply machine learning methods that can extract the most prevalent topics from the entire corpus of reports in an unbiased and data-driven manner.

Here, we use one of the most established free-text processing methods, Latent Dirichlet Allocation (LDA) [23], to identify prevalent topics from people's self-reported experiences of the COVID-19 pandemic during the peak of the first UK lockdown. First, we identified the most prevalent topics from questions probing the positive and negative impact of the pandemic. Next, we extracted the most common advice topics from the measures that participants recommended as helpful for coping with the challenges that the pandemic introduced to their daily lives. Finally, we tested the hypothesis that the impact that the pandemic had and the measures that people find most helpful vary substantially with age. We discuss the implications of the results for developing individually tailored and pragmatic digital therapies based on the collective lived experiences of the general population.

## 2. Methods

### 2.1. Recruitment

From 2 May 2020 (the time of maximal first UK lockdown), new participants in the Great British Intelligence Test study [15] were given the option to complete an extended section of the online questionnaire, which comprised pandemic-related items including three free-text fields.

A critical consideration when collecting free-text data is how to constrain the focus of the text to the general theme under investigation while ensuring that there is sufficient scope to express topics that are relevant to different people. Therefore, three broad questions were asked.

(1) ‘What has been most POSITIVE about the lockdown?’
(2) ‘What has been most NEGATIVE about the lockdown?’
(3) ‘What have you done that you would recommend to others because it has helped you during the lockdown?’

Additionally, all participants completed a sequence of cognitive tests and sociodemographic and mental health questionnaires, which form the focus of other research articles.

The study was promoted by advertisements on the BBC homepage and BBC2 Horizon website. Importantly, recruitment materials did not mention COVID-19, thereby reducing the risk of recruitment bias. To maximize the representativeness of the sample there were no inclusion/exclusion criteria. However, analyses here exclude data from participants under 16 years old, as they completed a briefer questionnaire and those who responded to the baseline questionnaire unfeasibly fast (less than 4 min), which would indicate that a person did not carefully read the questions. This threshold was determined prior to data analysis by consensus among the study team. The study was approved by the Imperial College Research Ethics Committee (17IC4009) and participants gave informed consent prior to participating.

### 2.2. Data collection

Data were collected via our custom Cognitron server system, which produces study-specific websites (https://gbws.cognitron.co.uk) and is hosted on the Amazon EC2. Questionnaires were programmed in JavaScript with HTML5 and were administered via personal computers, tablets and smartphones. The questionnaire was collected at the time of the first peak UK lockdown. Here, we analyse the following items. Free-text answers to the three questions outlined above. Self-reported age in years. Twelve items probing self-reported mood and anxiety symptoms were selected from the extensively validated Patient Health Questionnaire 2 and General Anxiety Disorder 7, respectively [24,25]. These ask about symptoms over the preceding two weeks, and each question is answered on a four-point scale, from 0 (not at all) to 3 (nearly every day). Additionally, we asked how many hours on average participants slept per night. A more complete description of the set of broader questionnaire items has been reported elsewhere [12].

### 2.3. Data processing and statistical analysis

Analyses were conducted in MATLAB R2020a using native functions for text processing and with a standard minimal preprocessing pipeline. Specifically, to maximize the use of data, and since some questionnaire responses were contingent on others, participants with missing data were retained. Free-text responses were first processed in the following steps. Entries under six characters in length were removed. Punctuation was erased. Stop words, non-words and words under three or over 14 letters long were removed. The remaining words were lemmatized (i.e. inflected forms of words were grouped so they could be analysed as a single item) and the documents tokenized (i.e. represented as collections of words).

Next, LDA was applied to extract common topics from the free text [23]. LDA is one of the most established methods for identifying commonly co-occurring combinations of words or ‘latent documents', which characterize the free-text observations in terms of the mixtures of topics from which they are comprised. Fine-tuning modelling functions can substantially impact on performance, which has implications for biasing results. Therefore, we used the native MATLAB implementation of LDA, which applies stochastic approximate variational Bayes [26], with all parameters on default settings.

A perennial question pertains to the optimal number of latent documents required to account for the observed text. This was estimated separately for each of the three free-text fields as follows. The participants were randomly split into two equal-sized train and test subsets, words occurring less than 10 times were removed, LDA models of different complexity (upper limit 80 topics) were fitted to the training subset and then evaluated against the test subset by taking the perplexity value, which quantifies fit of theoretical and observed topic word distributions. The process was repeated 20 times. The model complexity with mean lowest perplexity was identified and the LDA model retrained on all data at the corresponding number of topics. The word distributions and top 10 words and top 10 best-fitting text entries were examined for each topic in order to characterize them.

Each individual's free-text reports were classified according to the best-fitting topic, which was estimated by taking the topic that had the highest mixture coefficient for the corresponding text. Then, in order to test the hypothesis that the relevance of negative impact, positive impact and advice topics to people should vary with age, chi-squared tests were conducted on the frequencies of topics per age group at a 5-year precision (16–19, 20–24, 25–29, …, 75–79, 80+) and relative probabilities were plotted for evaluation.

## 3. Results

Between 2 May and 1 July 2020, 125 177 people across all ages undertook the study, with sampling heaviest towards the days immediately post-launch (counts per week: W1: 48 026; W2: 27 114; W3: 3960; W4: 1885; W5: 1119; W6: 762; W7: 477; W8: 260; W9: 213). After preprocessing, of the 83 816 adults 16 or older who completed the extended questionnaire, 48 315 opted to provide text in response to the positive impact of the pandemic; 48 482 provided text in response to the negative impact of the pandemic; and 44 376 provided advice free text. In total, 44 376 provided all three fields and 51 113 at least one field. The mean number of tokens (i.e. representing included lemmatized words) per document after preprocessing was similar for the three fields (positives: 7.9, negatives: 8.2 and advice: 7.7).

An overview of the analysed sample's demographic and other characteristics is presented in electronic supplementary material, supplement S1. The sample was diverse, spanning a wide age and education range, and inclusive of people of different sex, ethnicity, economic and occupational status.

Sampling bias for the extended questionnaire was evaluated with reference to scores on mental health items for the broader cohort versus those who undertook the extended questionnaire, and for those who completed the free-text fields versus those who chose not to. The key gauge of significance when dealing with big data is effect size. The cohort subsets had statistically significant differences in mental health scores; critically though, the differences were all of negligible scale (electronic supplementary material, supplement S2), indicating minimal sampling bias in this respect.

### 3.1. Self-reported negative impact of the pandemic

**Figure 1.**
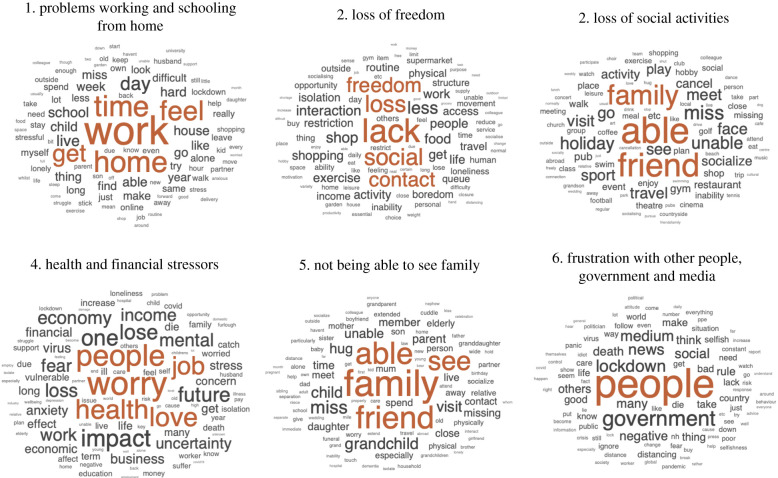
Word clouds showing the most common words per each of the six negative impact topics. Font size depicts word probabilities per topic. Topic labels are manually assigned based on the most probable words and top 10 best-fitting documents (electronic supplementary material, supplement S3).

Analysing mean perplexities in held out data to quantify model fit across the 20 iterations at each level of model complexity from 2 to 80 showed that six topics (table 1) gave the most optimal account of responses to the question: ‘what has been most NEGATIVE about the lockdown?’ from 49 482 participants. The top words for each topic and the best fitting (i.e. those with highest topic mixture) exemplar documents for each topic are presented in the electronic supplementary material, supplement S3 and figure 1. The most prevalent topic was ‘problems working and schooling from home’ (the best fit for the free text from 21.0% of participants). Next was ‘loss of social activities' (17.7%), followed by ‘not being able to see family (17.4%), ‘loss of freedom’ (16.5%), ‘health and financial stressors' (15.2%) and ‘frustration with inappropriate actions of other people, especially the government and media’ (12.4%).

**Table 1.** Topics from the LDA analysis of negative impact text.

mean mixture	most likely topic?		label
	*N*	%	
0.17	10 315	20.9	problems working and schooling from home
0.16	8138	16.5	loss of freedom
0.17	8777	17.7	loss of social activities
0.16	7517	15.2	health and financial stressors
0.18	8619	17.4	not being able to see family
0.15	6116	12.4	frustration with inappropriate actions of other people, especially the government and media
	49 482		total

### 3.2. Self-reported positive impact of the pandemic

**Figure 2.**
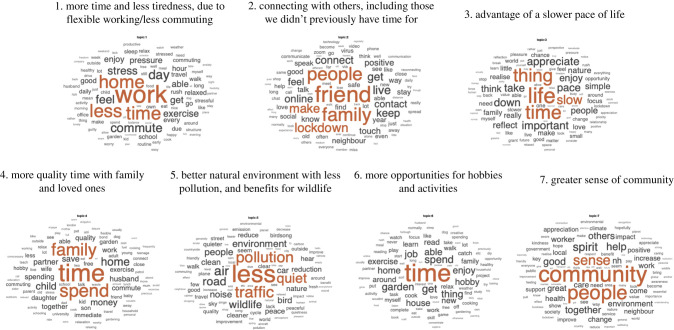
Word clouds showing the most common words per each of the seven positive impact topics. Font size depicts word probabilities per topic. Topic labels are manually assigned based on the most probable words and top 10 best-fitting documents (electronic supplementary material, supplement S4).

Analysing mean perplexities in held out data to quantify model fit across the 20 iterations at each level of model complexity from 2 to 80 showed that seven topics (table 2) gave the best account of responses to the question: ‘what has been most POSITIVE about the lockdown?’ from 48 315 participants. The top 10 words for each topic and the best fitting (i.e. those with highest topic mixture) exemplar documents for each topic are presented in figure 2 and electronic supplementary material, supplement S4.

**Table 2.** Topics from the LDA analysis of positive impact text.

mean mixture	most likely topic?		label
	*N*	%	
0.14	8542	17.7	more time and less tiredness, due to flexible working/less commuting
0.14	6948	14.4	connecting with others, including those we didn't previously have time for
0.13	5687	11.8	advantages of a slower pace of life
0.16	7751	16.0	more quality time with family and loved ones
0.15	7359	15.2	better natural environment with less pollution, and benefits for wildlife
0.15	6448	13.4	more opportunities for hobbies and activities
0.14	5580	11.6	greater sense of community.
	48 315		total

The most prevalent topic was ‘more time and less tiredness, due to flexible working / less commuting’ (17.7%). Next was ‘more quality time with family and loved ones' (16.0%), then ‘better natural environment with less pollution, and benefits for wildlife’ (15.2%), ‘connecting with others, including those we didn't previously have time for’ (14.4%), ‘more opportunities for hobbies and activities' (13.4%), ‘advantages of a slower pace of life’ (11.8%) and ‘greater sense of community’ (11.6%).

### 3.3. Self-reported practical strategies to maintain mental health and well-being

The perplexity estimates indicated that people's responses were far more diverse, as compared to the positive/negative impact of the pandemic, when answering the question ‘what have you done that you would recommend to others because it has helped you during the lockdown?’ 45 topics (table 3) provided the best account of responses from 44 376 people; two of which were excluded due to being thematically uninterpretable, leaving 43 topics. The most common topic was ‘help and care for others' (6.9%), followed by ‘maintain a regular healthy routine’ (4.8%), then ‘socialize via video conferencing technology’ (4.9%), ‘get outdoors for a walk and fresh air’ (4.3%), ‘do mindfulness activities' (4.1%) and ‘start new hobbies and interests' (3.3%). Top 10 exemplars for all topics are reported in electronic supplementary material, supplement S5 and top 10 words per topic are in electronic supplementary material, supplement S6.

**Table 3.** Topics from LDA analysis of pandemic advice free text.

mean mixture	most likely topic?		label
	*N*	%	
0.03	3043	6.9	HELP AND CARE FOR OTHERS
0.03	2124	4.8	MAINTAIN A REGULAR HEALTHY ROUTINE
0.04	2142	4.8	SOCIALIZE VIA VIDEO CONFERENCING TECHNOLOGY
0.03	1908	4.3	GET OUTDOORS FOR A WALK AND FRESH AIR
0.03	1823	4.1	DO MINDFULNESS ACTIVITIES
0.03	1200	2.7	MAKE SPACE FOR ‘ME TIME’ AND BE KIND TO YOURSELF
0.03	1482	3.3	START NEW HOBBIES AND INTERESTS
0.03	1224	2.8	TAKE THE OPPORTUNITY TO RELAX AND APPRECIATE THE SIMPLE THINGS IN LIFE
0.03	1199	2.7	THINK POSITIVE AND REMIND YOURSELF THAT THINGS COULD BE WORSE
0.03	1401	3.2	KEEP BOTH MIND AND BODY ACTIVE
0.03	1176	2.7	REMIND YOURSELF THAT THIS WILL NOT LAST FOREVER
0.03	1199	2.7	MAKE THE MOST OF THE TIME FOR ‘ODD JOBS’ AROUND THE HOME
0.03	1204	2.7	SPEND QUALITY TIME WITH FAMILY AND OUTDOORS
0.03	1314	3.0	LEARN NEW SKILLS
0.03	1264	2.9	DO CREATIVE, EXPRESSIVE ACTIVITIES
0.03	1150	2.6	MAINTAIN YOUR DAILY ROUTINES
0.02	987	2.2	ACCEPT THE THINGS YOU CANNOT CHANGE AND FOCUS ON CHANGING THE THINGS THAT YOU CAN
0.03	1221	2.8	KEEP IN TOUCH WITH FRIENDS AND FAMILY, USING TECHNOLOGY
0.02	1101	2.5	TAKE THE OPPORTUNITY TO PLAN A HEALTHIER DIET/EXERCISE REGIME
0.02	1098	2.5	LIMIT HOW MUCH TIME YOU SPEND READING/LISTENING/WATCHING NEGATIVE NEWS STORIES
0.02	804	1.8	TAKE A STEP BACK AND PRIORITIZE WHAT MATTERS TO YOU
0.02	916	2.1	AIM TO DO AT LEAST A BIT OF REGULAR EXERCISE
0.02	759	1.7	*Uninterpretable*
0.02	867	2.0	CONNECT WITH NATURE MORE
0.02	808	1.8	SET A FEW ACHIEVABLE GOALS TO DO EACH DAY
0.02	686	1.6	MAINTAINING A HEALTHY SLEEP WAKE CYCLE
0.02	755	1.7	TAKE THE TIME TO PLAN NICE HEALTHY MEALS
0.02	864	2.0	KEEP YOUR MIND ACTIVE
0.02	685	1.5	APPRECIATE THE NATURAL WORLD
0.02	670	1.5	DO VOLUNTEERING TO HELP OTHERS
0.02	712	1.6	SPEND MORE TIME ENJOYING MOVIES, READING, AUDIO BOOKS, AND MUSIC
0.02	680	1.5	MAINTAIN THE WORK LIFE BALANCE BY STRUCTURING YOUR DAY
0.02	695	1.6	DO ONLINE GROUP SOCIAL ACTIVITIES, SUCH AS QUIZZES AND MOVIE NIGHTS
0.02	561	1.3	PLAN AHEAD BUT NOT TOO RIGIDLY
0.02	383	0.9	*Uninterpretable*
0.02	499	1.1	MAKE MORE EFFORT TO KEEP IN TOUCH WITH LOVED ONES
0.02	461	1.0	MAKE A TO-DO LIST AND WORK THROUGH IT
0.01	348	0.8	USE THE TIME FOR THINGS YOU PUT OFF PREVIOUSLY
0.01	529	1.2	WALK IN THE COUNTRYSIDE
0.02	624	1.4	PLAY GAMES (E.G. BOARD GAMES, COMPUTER/VIDEO GAMES)
0.01	432	1.0	STAY AT HOME AND KEEP POSITIVE
0.01	407	0.9	FOLLOW THE COVID RULES, EVEN IF YOU MAY DISAGREE WITH SOME OF THEM
0.01	333	0.8	TRY TO AVOID NEGATIVE SOCIAL MEDIA STORIES
0.01	422	1.0	GROW YOUR OWN PLANTS/VEGETABLES AND NURTURE THEM
0.01	216	0.5	DO REGULAR PHYSICAL EXERCISE BECAUSE IT HELPS MENTAL AS WELL AS PHYSICAL HEALTH

### 3.4. Covariance of positive and negative impact topics with age

We tested the hypothesis that the ways in which people had been negatively and positively affected would covary with age. We classified participants according to their best-fitting negative and positive topics (i.e. the topic that had the highest mixture when accounting for each free-text response). Participants were grouped into age categories with a 5-year precision (16–19, 20–24, 25–29, 30–34, …, 75–79, 80+). Chi-squared tests confirmed that the distribution of best-fitting topics varied significantly across age categories (negative *X* = 2.11 × 10^3^ *p* < 0.0001; positives *X* = 4.48 × 10^3^, *p* > 0.0001). Further chi-squared tests were conducted to determine if each individual topic varied in prevalence across age groups (tables 4 and 5). Prevalence for each individual topic robustly covaried with age (all *p* < 0.001).

**Table 4.** Negative impact topic probabilities across age groups.

topic label	problems home working/ schooling	health and financial stressors	frustration with people/government/ media	not being able to see family	loss of freedom	loss of social activities
16–19	0.349	0.151	0.102	0.155	0.114	0.128
20–24	0.294	0.177	0.136	0.146	0.128	0.118
25–29	0.228	0.179	0.130	0.181	0.147	0.134
30–34	0.222	0.164	0.136	0.200	0.155	0.123
35–39	0.234	0.172	0.127	0.198	0.154	0.115
40–44	0.230	0.167	0.131	0.194	0.157	0.120
45–49	0.197	0.163	0.145	0.191	0.161	0.143
50–54	0.169	0.168	0.140	0.203	0.162	0.157
55–59	0.139	0.155	0.146	0.236	0.161	0.164
60–64	0.121	0.137	0.145	0.265	0.141	0.191
65–69	0.115	0.109	0.147	0.270	0.148	0.211
70–74	0.124	0.086	0.141	0.243	0.164	0.242
75–79	0.131	0.074	0.143	0.219	0.161	0.272
80+	0.171	0.075	0.128	0.190	0.202	0.234
chi-squared	1188	314	39	371	64	593
*p*	<0.0001	<0.0001	<0.0001	<0.0001	<0.0001	<0.0001
age correlation	−0.88	−0.85	0.56	0.70	0.72	0.90
max ratio	3.03	2.42	1.43	1.85	1.76	2.36

**Table 5.** Positive impact topic probabilities across age groups.

	more time/less tiredness	more quality time with family	advantages of a slower pace of life	more opportunity for hobbies/activities	connecting with others	greater sense of community	better natural environment
16–19	0.199	0.176	0.110	0.250	0.116	0.105	0.045
20–24	0.135	0.192	0.129	0.202	0.170	0.120	0.052
25–29	0.178	0.199	0.116	0.185	0.133	0.120	0.069
30–34	0.180	0.258	0.113	0.138	0.113	0.113	0.086
35–39	0.159	0.295	0.108	0.113	0.109	0.108	0.108
40–44	0.170	0.283	0.116	0.097	0.095	0.113	0.126
45–49	0.157	0.263	0.107	0.112	0.095	0.119	0.147
50–54	0.162	0.205	0.112	0.114	0.100	0.126	0.181
55–59	0.146	0.144	0.116	0.126	0.109	0.146	0.213
60–64	0.123	0.092	0.119	0.144	0.129	0.162	0.231
65–69	0.104	0.065	0.111	0.137	0.154	0.180	0.250
70–74	0.102	0.053	0.087	0.161	0.184	0.173	0.240
75–79	0.095	0.038	0.086	0.157	0.209	0.195	0.220
80+	0.109	0.029	0.104	0.123	0.251	0.188	0.196
chi-squared	288.839	2239.211	39.889	481.315	440.286	276.973	1575.683
*p*	<0.0001	<0.0001	<0.0001	<0.0001	<0.0001	<0.0001	<0.0001
age correlation	−0.85	−0.76	−0.61	−0.47	0.60	0.92	0.93
max ratio	2.09	10.25	1.49	2.57	2.66	1.86	5.53

**Figure 3.**
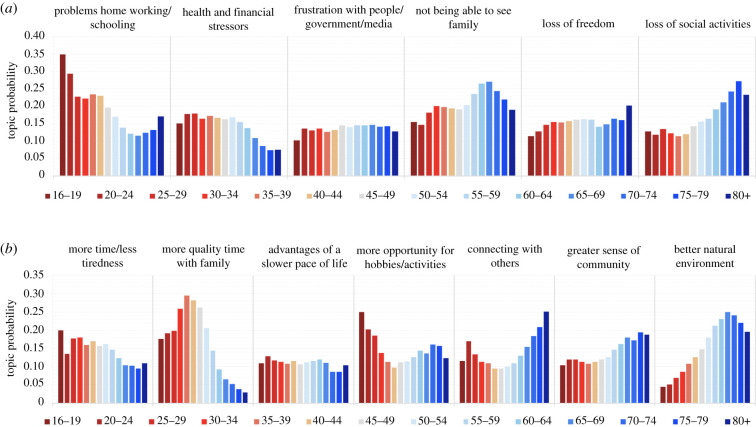
(*a*) The prevalence of the negative topics covaried significantly with age, with problems working or schooling from home being reported more often by teenagers and younger adults whereas the loss of social activities was more commonly reported by older adults. (*b*) Positive topics also showed substantial co-variance with age. Older adults were more likely to report being better connected with those they previously did not have time for, feeling there was a greater sense of community and noticing improvements in the environment. Younger adults and teenagers were more likely to report having more time/being less stressed and having more opportunity for hobbies and activities. People in their 30s and 40s were more likely to report more quality time with family.

Topic probability ratios were examined across age groups to determine the scale and nature of these associations (figure 3*a*). The negative topic that most strongly favoured young people was ‘problems working and schooling from home’, which was three times more likely in teenagers than people 60+; prevalence correlated with age group at *r* = −0.88. Conversely, the negative topic that most strongly favoured older adults was ‘loss of social activities', which was approximately twice as likely for adults in their 60s or above than those in their teens or twenties (topic probability versus age group correlation *r* = 0.9).

The positive topic that most strongly favoured young people was ‘more time and less tiredness, due to flexible working/less commuting’, which was 2.1 times more likely in teens than people 60+ (topic probability versus age group correlation *r* = −0.85) (figure 3*b*). Conversely, the topic ‘better natural environment with less pollution and benefits for wildlife’ was 5.5 times more likely in adults 80+ than teens (prevalence versus age group correlation *r* = 0.93). In accordance with past findings based on forced-choice responses, not all topics showed simple linear relationships with age. Most notably, the topic ‘more quality time with family and loved ones' was most common for people in their 30s and 40s, being more likely for late thirties relative to teens by 1.7 times and relative to 80+ by 10.3 times.

### 3.5. Covariance of advice topics with age

Finally, to test the hypothesis that age would affect the coping measures that people recommended during the pandemic, we conducted a chi-square test to determine whether the best-fitting advice topics for each participant covaried with the 5-year precision age categories. Overall, there was a significant association with age category (*x* = 2.75 × 10^3^ *p* = 2.36 × 10^−281^). When the probability of each topic was analysed individually across age groups (electronic supplementary material, supplement S7), only seven topics did not show significant covariance. Notably, one of these was the most prevalent topic ‘help and care for others'.

**Figure 4.**
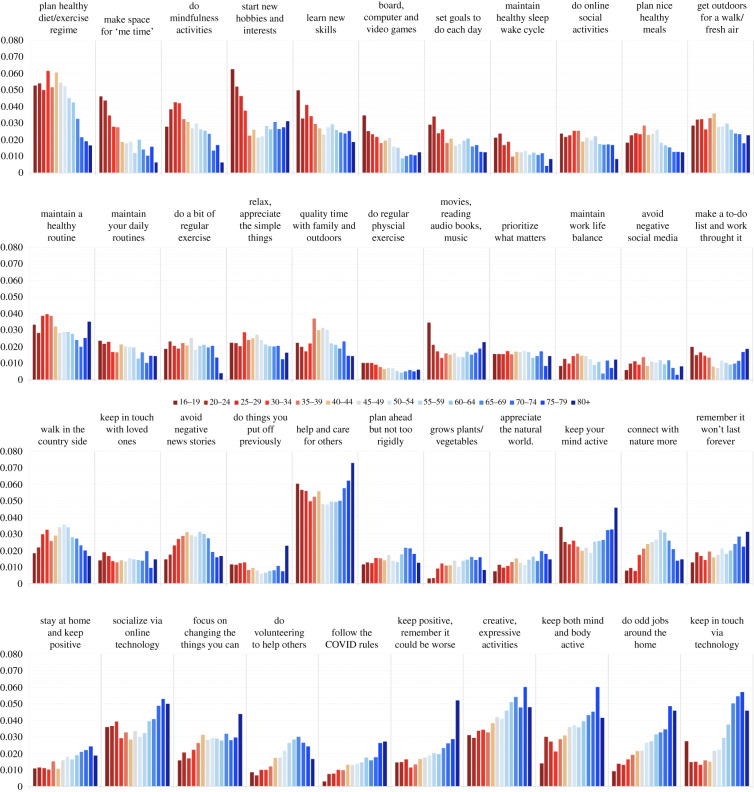
Advice topics are sorted according to correlation strength of prevalence across age groups. Many of the topics showed substantial covariance with age. Planning a healthy diet and/or exercise regime, making space for ‘me time’, doing mindfulness activities and learning new skills, interests or hobbies were all expressed substantially more often by teenagers and younger adults. Conversely, keeping in touch through technology, doing odd jobs around the home, keeping mind and body active, following COVID rules, keeping positive and engaging in creative/expressive activities were all substantially more likely to be reported by older adults. Avoiding negative news stories, walking in the countryside, connecting more with nature and spending more time outside with family were all more prevalent among people of middle working age. Helping and caring for others was one of the most prevalent advice topics across all age groups.

Closer inspection showed substantial probability ratios for many of the advice topics across age groups (figure 4). The strongest probability difference (electronic supplementary material, supplement S7) favouring older adults was for the topic ‘follow the COVID rules even if you may disagree with some of them’, which was 8.9 times more likely for people 80+ than teenagers and had an age × probability correlation of *r* = 0.95. Other topics strongly favouring older adults included ‘think positive and remind yourself that things could be worse’, ‘do creative expressive activities’, ‘keep both body and mind active’, ‘make the most of the time for odd jobs around the home’ and ‘keep in touch with friends and family using technology’.

The strongest probability difference favouring younger adults was for the topic ‘make space for ‘me time’ and be kind to yourself’, which was 7.4 times more likely for teenagers than people 80+ and had an age × probability correlation of *r* = −0.91. Other topics strongly favouring younger adults included, ‘do mindfulness activities’, ‘start new hobbies and interests’, ‘learn new skills’ and ‘play games (e.g. board games, computer/video games)’.

Not all topics that varied with age did so in a simple ordinal manner. Notably, some had the greatest prevalence for people of middle working age. These included the topics ‘take the opportunity to plan a healthier diet/exercise regime’, ‘spend quality time with family outdoors’, ‘walk in the countryside’, ‘connect with nature more’ and ‘limit how much time you spend reading/listening/watching negative news stories’.

## 4. Discussion

Our results provide a novel participant-driven perspective on the common positive and negative ways that the pandemic impacted on people's daily lives and of the diverse strategies used by people to cope with the pandemic (including lockdown). It was notable that in the LDA analyses, people's free-text descriptions of the negative and positive impact of the pandemic could be explained by a relatively small set of common topics. However, the prevalence of those topics varied substantially with age. This variability accords with our previous report based on large-scale cross-sectional analyses, where forced-choice responses were made to questionnaire items [12]. There, it was evident that different segments of society had been affected in different ways and that age was notable as a prominent covariate of both mental health symptom frequencies and aspects of change in daily life. Here, topics could be characterized as having either general relevance, or more commonly, greater relevance to older adults, younger adults, or those of middle working age.

Regarding teenagers and younger adults, the most prevalent negative topics related to problems with schooling or working from home and health or financial stressors, but notable positives included more time for hobbies/activities and having more time/being less tired. Conversely, older adults, particularly of retirement age, were more likely to worry about loss of social activities and loss of freedom but noted as positives a greater sense of community and connecting more with people who they previously had less time for. Older adults of working age were more likely to report not seeing family as the most prominent negative consequence of the pandemic whereas those of middle working age were more likely to report having more quality time with family, a difference that likely reflects those whose children have versus have not moved out. Frustration with the actions of others, especially government and the media, was a prevalent negative topic across all age groups, while advantages of a slower pace of life were noted as a positive with similar prevalence across all age groups.

Given this variability in how people of different ages were affected during the pandemic, it is unsurprising that the coping measures that they endorsed also covaried substantially with age. However, what was not expected was the diversity of such measures that people had identified. On a qualitative level, there appeared to be common themes that cut across the data-driven topics and that came together in different combinations. For example, planning ahead, setting goals and imposing structure on one's time were recurrent themes for coping across many of the advice topics. However, people recommended applying these strategies to a diversity of actions and pursuits. These interrelationships could form the basis of further study where advice topics are further classified relative to each other, This could be useful from an intervention perspective, enabling individuals to select from among pursuits/actions that are endorsed by people with similar profiles, and then further refining the search according to strategies that people recommend to help organize and implement them.

When inspecting the covariance of advice topics with age, common themes were evident in the patterns of covariance across age groups. These tended to pertain to the pursuits that formed the focus of the coping measures. Most notably, topics focused on physical exercise, mindfulness, ‘me time’ and the acquisition of new skills and hobbies were substantially more prevalent in teenagers and younger adults compared with retirees. Conversely, topics around the theme of keeping busy, be that with established hobbies, or jobs around the house, were substantially more prevalent in older adults, as was using modern information technologies to stay in touch. In general, people of middle working age were more likely to endorse spending time outside, be that to connect with nature, relax or have quality time with family. They were less likely to recommend finding ways to keep busy, such as via new hobbies, entertainment or making lists of jobs to do. The most prevalent topic overall was helping and caring for others. Interestingly, whereas that topic was somewhat lower for people of middle working age, it had broad relevance for all age groups. It also was somewhat counterbalanced by higher prevalence of the topic ‘do volunteering to help others'; this reinforces the view that advice topics might be thematically characterized according to what activities people have tried to achieve and how they recommend implementing them. A further example of this is that while younger adults were more likely to endorse mindfulness activities such as meditation or yoga, older adults were more likely to recommend positive thinking and being mindful that things could be worse and that the current global conditions will not last forever.

On a methodological level, this study demonstrated the largely untapped potential of combining free-text analysis methods from the machine learning field with a large-scale citizen science approach to engaging members of the general public in research en masse and collecting data in a directed but relatively unconstrained manner. This approach overcomes the issues of biased expectations and perceptions on the part of the researcher/surveyor, which may miss some of the most critical topics. It should be noted that we used one of the most established methods for distilling topics from free text and undertook no tuning of parameters. Despite this simplicity of analysis, the interpretability of the top words and top exemplars demonstrate that the method successfully identified topics that were coherent. The fact that topic prevalence covaried in a robust and interpretable manner with age provides further predictive validation that the modelling converged on an informative solution. Future studies should explore the relative performance of alternative topic modelling methods, especially the potential of approaches that can organize topics according to common themes.

Several limitations should be considered in relation to this study. First, we used an open citizen science approach to collect the data for this study. However, some groups promote the importance of random sampling methods [27]. Notably though, random sampling methods for surveys do not entirely eliminate bias as people are required to respond. Furthermore, we believe that the large cohort size and demonstrably high levels of inclusivity (i.e. including minority groups, and those with pre-existing mental and physical health conditions) mitigate the issue of sampling bias. Indeed, differences in mood self-assessment scores between those who did versus did not complete the free-text sections were of negligible scale. This indicates that our results are unlikely to paint an overly positive or negative picture of the general population's perspectives regarding the pandemic.

Second, we only examined here how topic prevalence covaried with age. This decision was made based on our previous work, where we showed that age is one of the most major determinants of differential pandemic impact [12], and because we had sampled a good spread of participants across a broad age range. Clearly age provides a good predictor of the positive, negative and advice topics applied here. Nonetheless, there is substantial future potential in examining covariance of topics with other contextual variables; for example, anxiety is also influenced by the health risk perception related to the probability of contracting the virus [28,29] such as the presence of particular disorders. Work and home context, gender, pregnancy and/or membership of minority groups also have been associated with the differential impact of the COVID-19 pandemic on mental health [12,30–33]. The current study focused on people aged 16 and older; but future work in younger people is also important, since they may have different experiences [5]. A challenge to address will be how to identify in a data-driven manner which from among the wide range of population variables has the greatest association with topic prevalence, or to organize people into sub-groups according to common conjunctions of many such variables.

Lastly, these data are cross-sectional in nature. We shall continue to collect data at six-month time points and intend to report on how the common topics evolve including with a more detailed assessment of identified vulnerable sub-groups. Future work with longitudinal mental health data in this cohort will examine whether the coping measures people have identified, as characterized by topic prevalence, are predictive of the change in mental health symptoms across time.

In summary, the findings here provide a rigorous demonstration of citizen science: by collecting free text from the general public at an unprecedented scale, we identified a cohesive set of topics regarding the impact of the pandemic, and how this can be mitigated, based on collective lived experience. All of the advice topics reported here must by definition have been endorsed by a substantial proportion of people within the UK general population in order to be evident within the LDA model. There is a self-evident tendency in the topics towards that which is broadly relevant and intrinsically feasible in terms of having either low or no financial cost and being demonstrably implementable in daily life. The fact that the topics vary in a predictable manner with a population variable, as exemplified by analyses on age, highlights the potential for developing online individually tailored digital advice interventions. We believe that this approach could have relevance not only within the pandemic, but as we move forwards through the recovery phase. In particular, while there has been much focus on the negative impact of the pandemic, many people report that some things have changed for the better [12]. Society could improve by learning from these lived positive experiences. Prominent examples include the additional time and flexibility that is afforded by working from home more and commuting less. Similarly, the reported increase in connectedness that communication technologies have afforded for older adults merits consideration by policy makers; as do the findings that ensuring access to green spaces and a greater focus on improving the environment were important. Overall, we suggest that public health strategies could be directly informed by such citizen science focused research approaches as they are inclusive and scientifically neutral.

## Data accessibility

Data may be made available for specific allowed purposes in pseudonymized format via data transfer agreement. Enquiries should be sent to the corresponding author.

The data are provided in the electronic supplementary material [34].
